# 3-*H*-[1,2]Dithiole as a New Anti-*Trypanosoma cruzi* Chemotype: Biological and Mechanism of Action Studies

**DOI:** 10.3390/molecules200814595

**Published:** 2015-08-12

**Authors:** Marcos Couto, Carina Sánchez, Belén Dávila, Valentina Machín, Javier Varela, Guzmán Álvarez, Mauricio Cabrera, Laura Celano, Beatriz Aguirre-López, Nallely Cabrera, Marieta Tuena de Gómez-Puyou, Armando Gómez-Puyou, Ruy Pérez-Montfort, Hugo Cerecetto, Mercedes González

**Affiliations:** 1 Grupo de Química Medicinal-Laboratorio de Química Orgánica, Facultad de Ciencias, Universidad de la República, Iguá 4225, Montevideo C.P. 11400, Uruguay; E-Mails: mcoutosire@gmail.com (M.C.); caarisanchez@hotmail.com (C.S.); BelenDav@gmail.com (B.D.); valeentiina.m@gmail.com (V.M.); jvarelaubillos@gmail.com (J.V.); guzmanalvarezlqo@gmail.com (G.Á.); mauriciocabreracedres@gmail.com (M.C.); 2 Laboratorio de Enzimología, Facultad de Ciencias, Universidad de la República, Iguá 4225, Montevideo C.P. 11400, Uruguay; E-Mail: lcelano@fcien.edu.uy; 3 Departamento de Bioquímica y Biología Estructural, Instituto de Fisiología Celular, Universidad Nacional Autónoma de México, Ciudad de México 04510, Mexico; E-Mails: bety.aguirre.lopez@gmail.com (B.A.-L.); ncabrera@ifc.unam.mx (N.C.); mtuena@ifc.unam.mx (M.T.G.-P.); apuyou@ifc.unam.mx (A.G.-P.); ruy@ifc.unam.mx (R.P.-M.)

**Keywords:** anti-*T. cruzi* activity, 3*H*-1,2-dithiole, triosephosphate isomerase, cruzipain, membrane sterol biosynthesis, ^1^H-NMR metabolomics

## Abstract

The current pharmacological Chagas disease treatments, using Nifurtimox or Benznidazole, show limited therapeutic results and are associated with potential side effects, like mutagenicity. Using random screening we have identified new chemotypes that were able to inhibit relevant targets of the *Trypanosoma cruzi*. We found 3*H*-[1,2]dithioles with the ability to inhibit *Trypanosoma cruzi* triosephosphate isomerase (*Tc*TIM). Herein, we studied the structural modifications of this chemotype to analyze the influence of volume, lipophilicity and electronic properties in the anti-*T. cruzi* activity. Their selectivity to parasites *vs.* mammalian cells was also examined. To get insights into a possible mechanism of action, the inhibition of the enzymatic activity of *Tc*TIM and cruzipain, using the isolated enzymes, and the inhibition of membrane sterol biosynthesis and excreted metabolites, using the whole parasite, were achieved. We found that this structural framework is interesting for the generation of innovative drugs for the treatment of Chagas disease.

## 1. Introduction

American trypanosomiasis, Chagas disease, is transmitted to humans by bites and concomitant defecation of different triatomine species, which carry the flagellate parasite *Trypanosoma cruzi* (*T. cruzi*) in their contaminated feces. It is an endemic disease that affects nearly 10 million people, generating health, economic and social problems in the affected countries [1]. Although the disease is native to Central and South America, nowadays both the population mobility between Latin America and the rest of the world, or the residence in endemic areas, have made it a worldwide problem.

Like other neglected diseases, it is a major health problem resulting from inadequate therapy and the lack of an effective vaccine [2,3]. Treatment of Chagas disease requires long-term dosage regimes with nifurtimox (Nfx; Lampit, Bayer Healthcare, Leverkusen) or benznidazole (Bnz; LAFEPE, Pernambuco), however, severe side effects often prompt the discontinuation of the treatment [4]. Among other relevant problems, these drugs exhibit significant mutagenic effects, and in some studies have been shown to be tumorigenic or carcinogenic [5].

**Figure 1:**
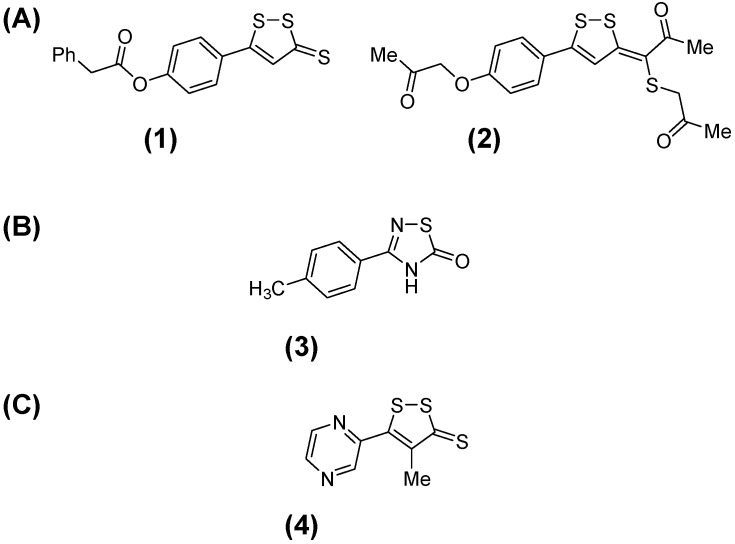
(**A**) Structures of the new hits, belonging to 3*H*-[1,2]dithiole chemotype and identified as *Tc*TIM inhibitors; (**B**) Structure of our best *Tc*TIM inhibitor described to date [11]; (**C**) Structure of oltipraz.

As part of our ongoing program in search of small molecular weight compounds that could provide leads in the design of new drugs for the treatment of Chagas disease [6], we undertook a massive screening for inhibitors of *Trypanosoma cruzi* triosephosphate isomerase (*Tc*TIM). *Tc*TIM is considered a potential target for anti-trypanosomal drugs [7,8,9]. This enzyme is involved in the glycolysis pathway of the parasite. We evaluated nearly 300 compounds from our in-house library against *Tc*TIM, identifying good inhibitors [10,11,12,13] and also hits with the ability to act as potential inhibitors, after further structural modifications. Of these hits we identified two compounds with an original framework in the context of anti-trypanosomal agents, the 3*H*-[1,2]dithiole heterocycles. These are compounds **1** and **2** (Figure 1A) [14], which, at 25 μM, had high percentages of *Tc*TIM inhibition, 33% and 48%, respectively. These compounds are structurally related to one of our best *Tc*TIM inhibitors, the [1,2,4]thiadiazol-5(4*H*)-one **3** (Figure 1B) [11].

On the other hand, the 3-*H-*1,2-dithiole heterocycle has been previously described as the framework responsible for schistosomicidal activity, e.g., in oltipraz (**4**, Figure 1C) [15].

However, according to our knowledge, there are no descriptions about the use of this kind of heterocycle as a drug pharmacophore for Chagas disease. 

The present study was undertaken in order to investigate the potential of different 3*H*-[1,2]dithiole derivatives as anti-*T. cruzi* agents and also to attempt to elucidate their mechanism(s) of action.

## 2. Results and Discussion

### 2.1. Synthesis of 3-H-[1,2]Dithiole Derivatives

**Figure 2:**
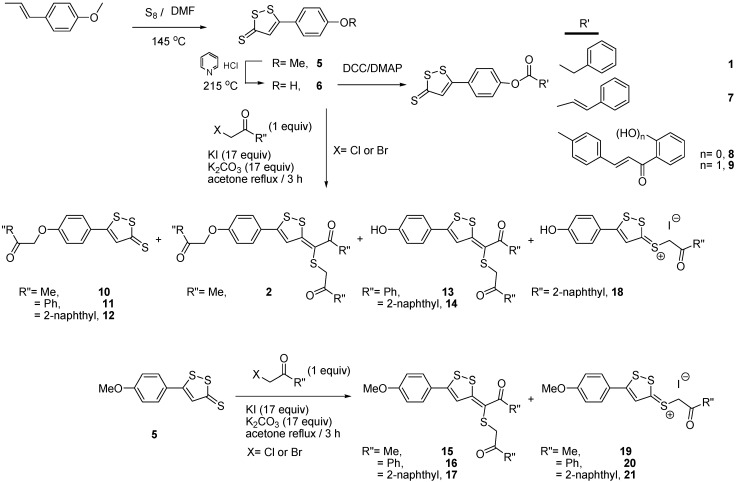
Synthetic procedures used to prepare the dithiole derivatives.

For the synthesis of the 3*H*-[1,2]dithiole derivatives **1**, **2** and **5**–**21** (Figure 2), we used anethole as starting material [16]. From the dithiolethione **6** we prepared esters **1**, and **7**–**9** via coupling with DCC/DMAP [14]. The (*E*)-3-[1-(alkylthio)propylidene]-3*H*-[1,2]dithioles **2**, and **13**–**17** were obtained using different α-haloketones in the presence of excess potassium iodide, according to previously reported protocols, starting from dithiolethiones **5** or **6** [17]. Additionally, in these reactions, products **10**–**12** and iodide salts **18**–**21** were also isolated. All the new compounds, **12**, **14**, **17**, **18**, and **21**, were characterized by ^1^H-NMR, ^13^C-NMR, COSY, HSQC, and HMBC experiments, and MS. According to H-H coupling constants and NOE-diff experiments, the compounds were obtained as the *E*-isomer around the alkenic moiety. The purity of the synthesized compounds was established by TLC and elemental analysis (C, H, N). Only compounds with analytical results within ±0.4 of the theoretical values were considered pure enough for biological testing.

### 2.2. In Vitro Biological Studies

To evaluate the anti-trypanosomal activity of the synthesized 3*H-*[1,2]dithioles we used *T. cruzi*, Tulahuen 2 strain (discrete typing unit, DTU, TcVI [18]) in the epimastigote form. Initially working at 25 µM, the IC_50_ was determined for compounds showing 70% of growth inhibition at this concentration (Table 1) [19]. To establish the selectivity of the compounds against the parasite, unspecific cytotoxicity against mammalian cells (J774.1 murine macrophages) was studied for the most relevant derivatives. The selectivity indexes (SI) were also determined (Table 1).

In general, the 3*H*-[1,2]dithiole-3-thiones had modest activity against *T. cruzi*, with phenols **6** and **9** showing the lowest IC_50_. Additionally, they had some level of parasite selectivity, SI ~ 2.0 (Table 1).

The iodide salts **18**–**21** were also inactive against *T. cruzi* (Table 1) probably as result of their higher hydrophilicities and a lower ability to cross the cellular membrane.

On the contrary, the 3-propylidene-3*H*-[1,2]dithioles displayed noteworthy activity against the epimastigotes of *T. cruzi*, being equipotent to the reference trypanosomicidal drugs Nfx and Bnz (IC_50,Nfx_ = 8.0 ± 1.0 μM, IC_50,Bnz_ = 7.0 ± 1.0 μM [19]). Again, phenols **13** and **14** were the most potent derivatives and, additionally, they had the highest selectivity (SI > 6.0). Consequently, the 3-(alkylthio)propylidene-3*H*-[1,2]dithiole structural framework emerges as a new hit for the development of anti-trypanosomal drugs. 

**Table 1:** Effects of the studied compounds against epimastigotes of *T. cruzi* (Tulahuen 2 strain) and murine macrophages (J774.1). The experiments were done in triplicate.

Family	Derivative	IC_50_ against *T. cruzi* (μM)	IC_50_ against Macrophages (μM)	SI	miLogP ^a^
**3*H*-[1,2]dithiole-3-thiones**	**1**	>25	- ^b^	-	4.66
	**5**	>25	-	-	3.57
	**6**	25.0 ± 1.0	51 ± 2	2.0	3.04
	**7**	>25	-	-	5.21
	**8**	>25	-	-	7.26
	**9**	25.0 ± 0.5	63 ± 2	2.5	7.20
	**10**	>25	-	-	3.12
	**11**	>25	-	-	4.72
	**12**	>25	-	-	5.91
**3-(alkylthio)propylidene-3*H*-[1,2]dithioles**	**2**	7.7 ± 1.4	24 ± 1	3.1	3.16
	**13**	5.5 ± 0.9	34.0 ± 0.5	6.2	6.28
	**14**	4.9 ± 1.1	63 ± 2	12.8	8.50
	**15**	5.9 ± 1.2	<24.0	<4.1	3.61
	**16**	>25	-	-	6.82
	**17**	>25	-	-	8.77
**[1,2]dithiolium iodide**	**18**	>25	-		3.25
	**19**	>25	-	-	4.07
	**20**	>25	-	-	2.60
	**21**	>25	-	-	3.78

^a^ LoP determined using Molinspiration online property calculation toolkit [20]. ^b^ -: not determined.

The lipophilicity seems to be partially related with the anti-*Trypanosoma cruzi* activity. The three most hydrophilic compounds, **6**, **10** and **20**, were inactive against the whole parasite. The same occurs with the most lipophilic compound, **17**. Similarly with the unspecific toxicity, the most lipophilic compounds **9** and **14** were the least cytotoxic. This physicochemical property is not the only related to the activity because no statistically significant correlation could be established.

### 2.3. Accessing the Mechanism of Action of the New Active Anti-T. cruzi Agents

In order to identify the potential modes of action of these new anti-*T. cruzi* agents, we made different kinds of experiments. Firstly, the compounds were tested as inhibitors of two relevant and validated targets of *T. cruzi*: the glycolytic enzyme triosephosphate isomerase (*Tc*TIM) and the protease cruzipain. Besides the studies on the isolated enzymes, we included two different types of experiments using the entire parasites. We studied the effects of the compounds on the pathways of membrane sterol biosynthesis. Also, we analyzed the modifications of the excreted metabolites.

#### 2.3.1. Inhibition of *Tc*TIM

In order to investigate if the 3*H*-[1,2]dithioles inhibit *Tc*TIM, we initially tested the inhibitory capacity of the compounds at a concentration of 200 μM and then at 25 μM. If the compound inhibited the enzymatic activity by more than 60% at 25 μM, the IC_50_ was determined (Table 2) [10,11,12].

**Table 2:** Inhibition of enzymatic activity of *Tc*TIM and cruzipain. The experiments were done in triplicate.

Family	Derivative	*Tc*TIM			Cruzipain	
		% Inhib_200_^a^	% Inhib_25_^a^	IC_50_ (μM)	% Inhib_100_^a^	IC_50_ (μM)
**3*H*-[1,2]dithiole-3-thiones**	**1**	77	33 ± 1.9	- ^b^	21.3 ± 0.3	-
	**5**	86	80 ± 2.3	1.2 ± 0.05	89.0 ± 0.5	4.0 ± 2.0
	**6**	71	35 ± 3.0	-	59.3 ± 0.3	-
	**7**	14	-	-	78.4 ± 0.6	-
	**8**	ns ^c^	-	-	58.0 ± 0.1	-
	**9**	50	-	-	77.9 ± 0.6	-
	**10**	70	61 ± 1.0	-	73.9 ± 0.1	-
	**11**	ns	-	-	96.3 ± 0.7	17.1 ± 3.6
	**12**	70	ni ^d^	-	91.5 ± 0.6	21.6 ± 6.1
**3-(alkylthio)propylidene-3*H*-[1,2]dithioles**	**2**	72	48 ± 4.0	-	91.3 ± 2.8	15.5 ± 2.7
	**13**	86	75 ± 6.0	3.35 ± 0.14	61.0 ± 0.3	-
	**14**	70	38 ± 3.5	-	66.5 ± 0.3	-
	**15**	86	62 ± 3.0	-	85.4 ± 0.2	15.1 ± 2.5
	**16**	77	63 ± 0.1	7.53 ± 0.10	62.3 ± 0.3	-
	**17**	81	52 ± 2.5	-	82.6 ± 0.6	-
**[1,2]dithiolium iodide**	**18**	70	75 ± 0.3	11.03 ±0.03	77.1 ± 0.1	-
	**19**	95	73 ± 3.2	3.26 ± 0.05	90.5 ± 0.7	11.3 ± 0.6
	**20**	ns	-	-	72.0 ± 0.1	-
	**21**	65	-	-	90.2 ± 0.6	17.7 ± 3.9

^a^ % Inhib: percentage of enzymatic inhibition at 200, 100 or 25 μM. ^b^ -: not determined. ^c^ ns: not studied. ^d^ ni: not inhibition.

Different levels of *Tc*TIM inhibition were observed with the compounds. In each compound family, one or two derivatives displayed an excellent ability to inhibit this enzyme, e.g., the [1,2]dithiole-3-thione **5**, the 3-propylidene[1,2]dithioles **13** and **16**, and [1,2]dithiolium salts **18** and **19**. The best inhibitor was thione **5**, which had the same potency as **3** (Figure 1) [11]. Interestingly, excluding derivative **13**, no relationship between anti-epimastigote activity, of the most active compounds, and inhibition of *Tc*TIM was observed. In the case of the 3-propylidene[1,2]dithiole **13**, the IC_50_ against the whole parasite and *Tc*TIM are very similar suggesting that the mechanism of action of this compound could be the inhibition of this enzyme.

However, it was not possible to establish a clear relationship between anti-epimastigote activities and the *Tc*TIM inhibition activity for the whole series of studied compounds.

#### 2.3.2. Inhibition of Cruzipain

Initially, we tested the dithioles as inhibitors of the protease cruzipain from *T. cruzi* at a concentration of 100 μM. If the compound inhibited more than 85% of enzyme activity at this dose, the IC_50_ was determined (Table 2) [21].

In each family of compounds, two or three derivatives inhibit cruzipain in the micromolar range ([1,2]dithiole-3-thione **5**, **11** and **12**, 3-propylidene[1,2]dithioles **2** and **15**, and [1,2]dithiolium salts **19** and **21**). The best inhibitor was thione **5** which displayed low micromolar IC_50_ value. With the exception of compounds **2** and **15** which inhibit *T. cruzi* epimastigotes and cruzipain with IC_50_ values of the same order of magnitude (two-fold higher for cruzipain), no relationship between anti-epimastigote activity and cruzipain inhibition was observed.

Interestingly, [1,2]dithiole-3-thione **5** and 1 [1,2]dithiolium salt **19**, which are inactive against the whole parasite, were able to inhibit both *Tc*TIM and cruzipain in the low micromolar range. 

#### 2.3.3. Inhibition of Membrane Sterol Biosynthesis

**Figure 3:**
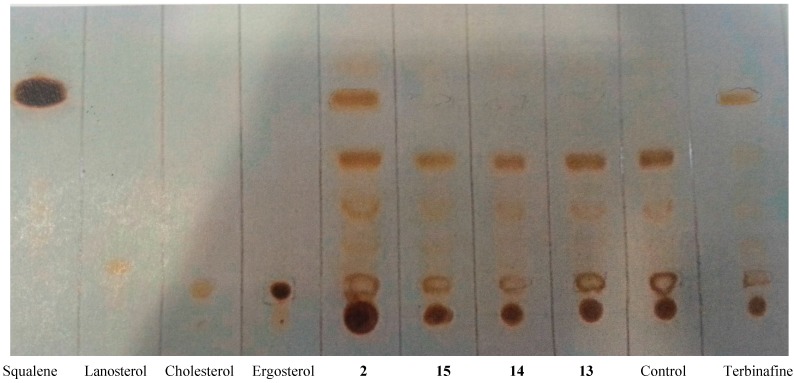
Example of a TLC for the study of changes in membrane sterols of *T. cruzi*. The lane labeled Control had untreated parasites. Terbinafine was used as a control of an accumulator of squalene for *T. cruzi*.

We analyzed the capacity of the most active compounds against epimastigotes (3-propylidene[1,2]dithioles **2**, and **13**–**15**) to inhibit any of the enzymes involved in the biosynthesis of membrane sterols. To do so, we analyzed the accumulation or depletion of some intermediates or final products of this biochemical pathway. Qualitative analyses were performed by thin layer chromatography (TLC) [22]. In these experiments, derivative **2** was able to accumulate squalene and deplete ergosterol (Figure 3), as did the positive control terbinafine, a well-known antifungal with anti-*T. cruzi* activity. According to these results, a possible target might be the enzyme squalene-2,3-epoxidase, which catalyzes the conversion of squalene into lanosterol [23]. Compared to control, the rest of the compounds studied did not show any changes in the levels of the sterols and intermediates analyzed.

#### 2.3.4. ^1^H-NMR Metabolomic Studies

To gain insight into the changes promoted by the active 3-propylidene[1,2]dithioles **2** and **14** in some biochemical pathways, the metabolites excreted by the parasite using ^1^H-NMR spectroscopy were analyzed [22]. Changes in the excreted metabolites by *T. cruzi* Y strain (DTU Tc II, [24]), when the parasite cells were exposed to a bioactive compound, could be indicative of the modification of the biochemical pathway(s) by the agents [25,26]. Spectra of the cell-free milieu of treated parasites were compared with those of the untreated *T. cruzi-*free milieu as the control. We focused mainly on the changes of excreted salts of the carboxylic acids, lactate (Lac), acetate (Ace), pyruvate (Pyr), and succinate (Succ) and the amino acids, alanine (Ala) and glycine (Gly), among the most relevant modified metabolites. 

Clearly, derivative **2** significantly decreased the levels of excreted Gly and Pyr. However, derivative **14** did not modify the excreted catabolites when compared to the control (Table 3).

**Table 3:** Concentrations of metabolites excreted, carboxylic acids and amino acids, in the metabolomic studies using ^1^H-NMR (for details see Experimental Section). Each run was made in triplicate.

Compound ^a^/	Gly	Succ	Pyr	Ace	Ala	Lac
Metabolite ^b^						
2	2.11 ± 0.11 ^c^	5.83 ± 0.24	8.00 ± 0.35	23.28 ± 0.86	17.76 ± 0.61	8.73 ± 0.37
14	2.48 ± 0.14	7.34 ± 0.65	9.47 ± 0.53	26.41 ± 1.44	20.65 ± 1.27	10.41 ± 0.58
Control ^d^	2.48 ± 0.11	6.64 ± 0.18	8.89 ± 0.27	26.06 ± 2.07	21.38 ± 1.15	9.69 ± 0.68

^a^ Working at the IC_50_ × 2 for each fraction. ^b^ The concentrations of the metabolites were calculated using DMF as the internal standard (for details see Experimental Section). ^c^ Statistically significant changes (*p* < 0.05, Student’s *t*-test) are shown in bold characters. ^d^ Untreated parasite.

**Figure 4:**
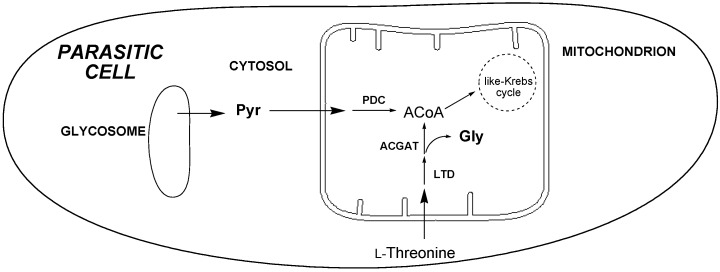
Possible site of action for derivative **2** in the biochemical pathways, according to the ^1^H-NMR metabolomic studies [27].

In the metabolic pathways of *T. cruzi* the depletion of both Gly and Pyr, as occurs with the 3-propylidene[1,2]dithiole **2**, probably indicates that some enzymes involved in the metabolism of L-threonine in the mitochondrion could be the potential targets of this compound [27]. The inhibition of L-threonine dehydrogenase (LTD) or acetyl-CoA:glycine *C*-acetyltransferase (ACGAT) could not only produce the reduction of excreted Gly, but also the production of acetyl-CoA (ACoA), in the mitochondrion, which are further used in the like-Krebs cycle. Consequently, if the ACoA is reduced, by inhibition of LTD or ACGAT, the parasite needs to use the Pyr entering the organelle from the cytosol and transform it to ACoA via the pyruvate dehydrogenase complex (PDC), to maintain the energetic requirements (Figure 4). This fact produces a concomitant depletion of excreted Pyr. In order to confirm this metabolic pathway, more experiments must be done, like the inhibition of LTD o ACGAT.

## 3. Experimental Section

### 3.1. General

Reagents were purchased from Aldrich and used without further purification. Melting points were performed using an Electrothermal Engineering Ltd melting point apparatus (Rochford, UK), and the results were uncorrected. ^1^H-NMR and ^13^C-NMR spectra were recorded in the indicated solvent with a Bruker DPX 400 MHz spectrometer (Bruker, Rheinstetten, Germany). Chemical shifts are quoted in parts per million downfield from TMS and the coupling constants are in Hertz. Structural assignments were corroborated by HMBC and HSQC experiments. All solvents were dried and distilled prior to use. All the reactions were carried out in a nitrogen atmosphere. Reactions were monitored by TLC using commercially available precoated plates (Merck Kieselgel 60 F254 silica, Darmstadt, Germany) and the developed plates were examined under UV light (254 nm) or as iodine vapor stains. Column chromatography was performed using a 200 mesh silica gel. To determine the purity of the compounds, microanalyses were done on a Fisons EA 1108 CHNS–O instrument (Ipswich, UK) from vacuum-dried samples and were within ± 0.4 of the values obtained by calculating their compositions. Compounds **1**, **2**, **5**–**11**, **13**, **15**, **16**, **19** and **20** were prepared following synthetic procedures previously reported [14,16,17].

### 3.2. General Synthetic Procedure for Compounds ***12***, ***14***, ***17***, ***18***, and ***21***

In a 25-mL round-bottom flask, under nitrogen atmosphere, a mixture of 2-bromo-2′-acetonaphthone (1 equiv.), **5** or **6** (1 equiv.), K_2_CO_3_ (17 equiv.), KI (17 equiv.) and anhydrous acetone (10 mL per 0.2 mmol of naphthone) was stirred at reflux for 5 h. The solvent was then evaporated to dryness, and the resulting residue was suspended in EtOAc (35 mL) and washed with 35 mL of water. Two extractions of the water were performed with EtOAc (2 × 35 mL), and the organic layers were dried over Na_2_SO_4_, evaporated *in vacuo*, and purified by column chromatography (SiO_2_, hexane:AcOEt, 7:3) to give the desired products **12**, **14**, and **18** from compound **6**, and **17**, and **21** from compound **5**.

*5-[4-(2-Oxo-2-naphthylethyloxy)phenyl]-3H-[1,2]dithiole-3-thione* (**12**). Yield: 29%; orange solid, mp: 130–133 °C; ^1^H-NMR (400 MHz, CDCl_3_) δ: 8.56 (s, 1H), 8.06 (dd, *J* = 8.6, 1.7 Hz, 1H), 8.03 (d, *J* = 7.8 Hz, 1H), 7.99 (d, *J* = 8.6 Hz, 1H), 7.94 (d, *J* = 8.2 Hz, 1H), 7.71–7.69 (m, 2H), 7.64 (d, *J* = 8.8 Hz, 3H), 7.41 (s, 1H), 7.07 (d, *J* = 8.9 Hz, 2H), 5.55 (s, 2H); ^13^C-NMR (100 MHz, CDCl_3_) δ: 215.3, 193.3, 172.7, 161.3, 136.1, 135.0, 132.4, 131.5, 130.0, 129.9, 129.7, 129.2, 128.7, 128.0, 127.5, 127.3, 125.1, 125.0, 124.9, 123.4, 115.8, 70.6. *Anal.* calc. for C_21_H_14_O_2_S_3_: C 63.9%, H 3.6%, S 24.4%. Found: C 64.0%, H 3.7%, S 24.3%.

*(E)-5-(4-Hydroxyphenyl)-3-[2-oxo-1-(2-oxo-2-naphthylethylthio)-2-naphthylethylidene]-3H-[1,2]dithiole* (**14**). Yield: 26%; orange oil; ^1^H-NMR (400 MHz, CDCl_3_) δ: 8.32 (s, 1H), 8.13 (s, 1H), 8.01 (s, 1H), 7.88 (dd, *J* = 8.4, 1.7 Hz, 1H), 7.81 (d, *J* = 8.8 Hz, 1H), 7.74 (d, *J* = 8.6 Hz, 2H), 7.69 (d, *J* = 8.0 Hz, 2H), 7.66 (d, *J* = 1.8 Hz, 1H), 7.56 (d, *J* = 8.4 Hz, 2H), 7.53 (d, *J* = 1.2 Hz, 1H), 7.49–7.43 (m, 3H), 7.33 (d, *J* = 8.7 Hz, 2H), 6.74 (d, *J* = 8.7 Hz, 2H), 5.60 (s, 1H), 3.79 (s, 2H); ^13^C-NMR (100 MHz, CDCl_3_) δ: 195.1, 189.7, 188.4, 167.0, 158.6, 135–115 (27 C), 42.2. *Anal.* calc. for C_33_H_22_O_3_S_3_: C 70.4%, H 3.9%, S 17.1%. Found: C 70.1%, H 3.6%, S 17.3%.

*5-(4-Hydroxyphenyl)-3-naphthoylmethylthio-[1,2]dithiolium iodide* (**18**). Yield: 13%; red oil; ^1^H-NMR (400 MHz, CDCl_3_) δ: 8.51 (s, 1H), 8.27 (d, *J* = 0.9 Hz, 1H), 8.11 (s, 1H), 7.81 (s,1 H), 7.80 (s, 1H), 7.56–7.50 (m, 5H), 7.01 (d, *J* = 8.6 Hz, 2H), 5.32 (s, 1H), 4.08 (s, 2H); ^13^C-NMR (100 MHz, CDCl_3_) δ: 188.1, 177.4, 168.2, 159.4, 140.0–110.0 (16 C), 33.5. *Anal.* calc. for C_21_H_15_IO_2_S_3_: C 48.3%, H 2.9%, S 18.4%. Found: C 47.9%, H 2.6%, S 18.3%.

*(E)-5-(4-Methoxyphenyl)-3-[2-oxo-1-(2-oxo-2-naphthylethylthio)-2-naphthylethylidene]-3H-[1,2]dithiole* (**17**). Yield: 20%; yellow oil; ^1^H-NMR (400 MHz, CDCl_3_) δ: 8.34 (s, 1H), 8.15 (s, 1H), 8.03 (s, 1H), 7.91 (dd, *J* = 8.4, 1.7 Hz, 1H), 7.83 (d, *J* = 7.2 Hz, 1H), 7.76 (dd, *J* = 9.0, 3.1 Hz, 2H), 7.71 (d, *J* = 8.0 Hz, 2H), 7.68 (d, *J* = 1.8 Hz, 1H), 7.58 (d, *J* = 8.4 Hz, 2H), 7.55 (s, 1H), 7.52–7.45 (m, 2 H), 7.35 (d, *J* = 8.7 Hz, 2H), 6.76 (d, *J* = 8.7 Hz, 2H), 4.00 (s, 3H), 3.81 (s, 2H); ^13^C-NMR (100 MHz, CDCl_3_) δ: 195.5, 185.2, 181.4, 170.0, 161.3, 139.4–114.5 (25 C), 111.2, 106.4, 55.4, 42.3. *Anal.* calc. for C_34_H_24_O_3_S_3_: C 70.8%, H 4.2%, S 16.7%. Found: C 70.5%, H 3.9%, S 16.6%.

*5-(4-Methoxyphenyl)-3-naphthoylmethylthio[1,2]dithiolium iodide* (**21**). Yield: 17%; red oil; ^1^H-NMR (400 MHz, CDCl_3_) δ: 8.57 (s, 1H), 8.12 (dd, *J* = 8.6, 1.7 Hz, 1H), 8.00 (d, *J* = 6.8 Hz, 1H), 7.95 (d, *J* = 8.5 Hz, 1H), 7.92 (s, 1H), 7.69 (d, *J* = 8.9 Hz, 2H), 7.67 (s, 1H), 7.58–7.57 (m, 1H), 7.56 (s, 1H), 7.01 (d, *J* = 8.8 Hz, 2H), 4.04 (bs, 2H), 3.91 (s, 3H); ^13^C-NMR (100 MHz, CDCl_3_) δ: 190.1, 178.4, 167.2, 159.7, 140.0–110.0 (16 C), 54.9, 33.8. *Anal.* calc. for C_22_H_17_IO_2_S_3_: C 49.2%, H 3.2%, S 17.9%. Found: C 48.9%, H 2.9%, S 17.6%.

### 3.3. Anti-T. cruzi Test in Vitro

We used epimastigotes of the Tulahuen 2 strain (DTU, Tc VI) growing in an axenic milieu (BHI-Tryptose). Cells from a 5–7-day-old culture were inoculated in fresh culture milieu to give an initial concentration of 1 × 10^6^ cells/mL. The absorbance at 600 nm of the cells in culture was measured every day. At day 5, the milieu was inoculated with different quantities of the compounds from a stock solution in DMSO (DMSO concentration in the culture milieu never exceeded 0.4%). The control was made in the presence of 0.4% DMSO and in the absence of compounds. Each concentration of compound was evaluated in duplicate. At day 5, the absorbance of the culture was measured and related to the control. The IC_50_ value was taken as the concentration of drug needed to reduce the absorbance ratio to 50%.

### 3.4. Unspecific in Vitro Cytotoxicity of Mammalian Cells

J774.1 murine macrophage cells (ATCC, Manassas, VA, USA) were grown in DMEM culture milieu containing 4 mM l-glutamine and supplemented with 10% heat-inactivated fetal calf serum. The cells were seeded in a 96-well plate (5 × 10^4^ cells in 200 mL culture medium) and incubated at 37 °C in a 5% CO_2_ atmosphere for 48 h, to allow cell adhesion prior to drug testing. Afterwards, cells were exposed for 48 h to the compounds (25–400 μM) or vehicle for control (0.4% DMSO), and additional controls (cells in medium) were used in each test. Cell viability was then assessed by measuring the mitochondria-dependent reduction of MTT (3-(4,5-dimethylthiazol-2-yl)-2,5-diphenyltetrazolium bromide) to formazan. For this purpose, MTT in sterile PBS (0.2% glucose) pH 7.4 was added to the macrophages to achieve a final concentration of 0.1 mg/mL and the cells were incubated at 37 °C for 3 h. After removing the medium, formazan crystals were dissolved in 180 μL of DMSO and 20 μL of MTT buffer (0.1 M glycine, 0.1 M NaCl, 0.5 mM EDTA, pH 10.5) and the absorbance at 560 nm was measured. The IC_50_ was defined as the drug concentration at which 50% of the cells were viable, relative to the control (no drug added), and was determined by analysis using OriginLab8.5^®^ sigmoidal regression (% of viable cells *vs.* logarithm of the compound concentration). Samples were performed in triplicate.

### 3.5. Inhibition of TcTIM

Expression and purification of protein: *Tc*TIM was expressed in *Escherichia coli* and purified as described in the literature [28]. After purification, the enzyme, dissolved in 100 mM triethanolamine, 10 mM EDTA and 1 mM dithiothreitol (pH 8), was precipitated with ammonium sulfate (75% saturation) and stored at 4 °C. Before use, extensive dialysis against 100 mM triethanolamine/10 mM EDTA (pH 7.4) was performed. Protein concentration was determined by absorbance at 280 nm (ε = 36,440 M^−1^·cm^−1^).

Enzymatic activity assays. Enzymatic activity was determined following the conversion of glyceraldehyde 3-phosphate into dihydroxyacetone phosphate [7]. The decrease in absorbance at 340 nm was followed in a multicell Cary spectrophotometer at 25 °C. The reaction mixture (1 mL, pH 7.4) contained 100 mM triethanolamine, 10 mM EDTA, 0.2 mM NADH, 1 mM glyceraldehyde 3-phosphate, and 0.9 units of α-glycerol phosphate dehydrogenase. The reaction was initiated by addition of 5 ng/mL of the *Tc*TIM.

For inactivation studies, *Tc*TIM was incubated at a concentration of 5 mg/mL in a buffer containing 100 mM triethanolamine, 10 mM EDTA, pH 7.4 and 10% of DMSO at 36 °C. The mixture also contained the compounds at the indicated concentrations. Compounds were dissolved in DMSO. After 2 h, 1 mL was withdrawn and added to 1 mL of reaction mixture for the activity assay. None of the molecules tested here affected the activity of α-glycerol phosphate dehydrogenase, the enzyme used for trapping the product. 

The IC_50_ value was taken as the concentration of drug needed to reduce the enzymatic activity to 50%. The experiments were performed in triplicate.

### 3.6. Inhibition of T. cruzi Cruzipain

Cruzipain was purified to homogeneity from epimastigotes of the Tulahuen 2 strain by ConA-Sepharose affinity chromatography, as previously described [29]. Cruzipain (2.5 μM ε = 58,285 M^−1^·cm^−1^) was incubated in 50 mM acetate buffer pH 5.5 with 50 mM DTT and 100 μM inhibitor was added, shaking the solution for 15 min at 27 °C. The derivatives were added diluted in DMSO, and the controls contained the same solvent concentration. The concentration of DMSO never exceeded 1% in the reaction medium. E-64 was used as a positive control of inhibition. Then, the fluorogenic substrate Z-Phe-Arg-AMC (100 μM) was added and the fluorescence was measured during 10 min at intervals of 3 s (excitation at 350 nm and emission at 460 nm) using a Varioskan Flash Spectrophotometer. From the slope of the negative control, we calculated the total (100%) enzyme activity, while the slopes obtained in the presence of the compounds yielded the percentage of remaining enzyme activity. The percentage of enzyme inhibition was determined as 100% of remaining enzyme activity. The experiments were done in duplicate.

### 3.7. Inhibition of Membrane Sterol Biosynthesis

The parasites, epimastigotes of Y strain of *T. cruzi* (DTU Tc II), were grown in 6 mL of BHT milieu in culture bottles. Compounds at their respective IC_50_ were incubated for 72 h at 28 °C with the parasite, starting from a parasitic load of 10 million per mL. A negative control was performed with parasites in the absence of compounds. The parasites in the positive control were incubated with terbinafine (IC_50_ = 44.7 μM), which has proven inhibitory activity on membrane sterol biosynthesis. The extraction of membrane sterols was performed on completion of the incubation. For this purpose, the content of the culture bottle was centrifuged at 3000 rpm for 10 min, the supernatant was discarded and the pellet was suspended in sodium phosphate buffer solution (6 mL, 0.05 M, pH 7.4). Then it was centrifuged again at 3000 rpm for 10 min and the supernatant was discarded. The resulting pellet was resuspended in a chloroform/methanol (2:1) mixture (5 mL) and the suspension was kept at 4 °C for 12 h. Then, a saturated NaCl solution (5 mL) was added and the mixture extracted once with chloroform (3 mL) and once with hexane (3 mL) with care to avoid taking any aqueous phase. The extracted volume was applied to a silica gel TLC plate. Chromatography was performed eluting with hexane, two runs to identify squalene, and once with hexane/EtOAc (8:2) for ergosterol. The spots on the plate were revealed under ultraviolet light or by exposure to iodine vapors. Also, controls and commercial samples of ergosterol, lanosterol, cholesterol and squalene were run on the TLC plate [30]. The experiment was done in triplicate.

### 3.8. ^1^H-NMR Study of the Excreted Metabolites

For the ^1^H-NMR spectroscopic studies, *T. cruzi* cells (Y strain, DTU Tc II) treated for 2 days, with each studied compound at concentrations corresponding to 2 × IC_50_ values (5 mL) were centrifuged at 1500*× g* for 10 min at 4 °C. The pellet was discarded, and the parasite-free supernatant was stored at −20 °C until use. Before measuring, DMF (0.1 mL, 10 mM) as the internal standard and D_2_O (0.1 mL) were added to the supernatant (0.3 mL). ^1^H-NMR experiments were recorded at 20 °C on a Bruker Advance DPX-400 spectrometer (Bruker), operating at 400.132 MHz, with a 5 mm broadband inverse geometry probe. The acquisition parameters included: 90° pulse (zgpr, advance-version v 1.7.10.2, 1D sequence with f1 presaturation), 128 scans, and spectral width of 14.983 ppm. The acquisition time was 1.3664 s. Signal intensities were calculated by performing appropriate baseline corrections and then integrating the area under each of the resonances using MestRe-C NMR software. Spectra were analyzed using the Topspin 1.3 software package. The spectra were registered with water suppression in 5 mm NMR (Aldrich, St. Louis, MO, USA) sample tubes. The chemical displacements used to identify the respective metabolites were previously confirmed by adding each analyzed metabolite to the studied supernatant, as well as by the study of a control solution with 4 μg/mL of each metabolite in buffer (phosphate, pH = 7.4). Each run was done at least in triplicate and the Student t test was used to analyze the significance of the changes. The chemical shifts (δ, ppm) and multiplicity of the analyzed catabolites were: Ala (alanine), 1.316, d; Lac (lactate), 1.466, d; Ace (acetate), 1.904, s; Pyr (pyruvate), 2.357, s; Succ (succinate), 2.392, s; Gly (glycine), 3.547, s [31].

## 4. Conclusions

**Figure 5:**
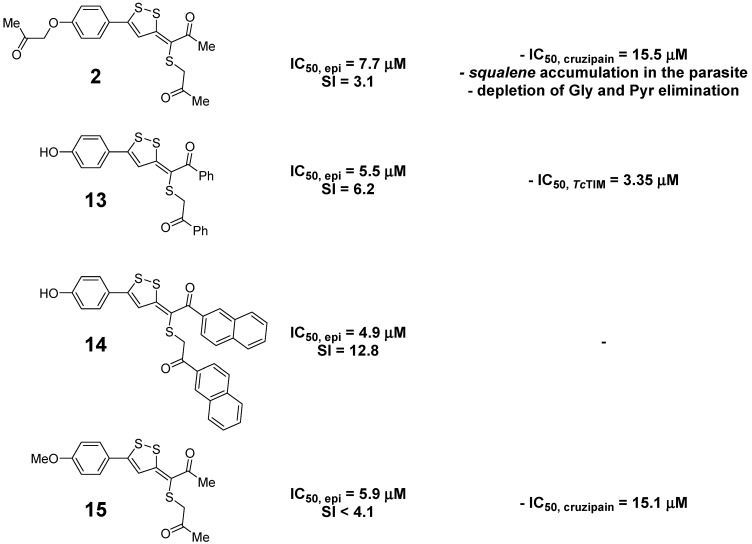
Summary of the identified 3-(alkylthio)propylidene-3*H*-[1,2]dithiole derivatives as the best anti-*T. cruzi* agents.

We have identified four derivatives, **2**, **13**, **14**, and **15**, with good anti-*T. cruzi* activity and selectivity profiles with different kinds of effects on the biochemical pathways of the parasite (Figure 5). 

Specifically, 3-(alkylthio)propylidene-3*H*-[1,2]dithiole **2** could be considered as a multitarget anti-*T. cruzi* agent showing modest *Tc*TIM and cruzipain inhibitions, at the micromolar level, and effects on the membrane sterol biosynthesis and on some mitochondrial enzymes. The dithiole **2** could be classified as a symbiotic agent [32].

On the other hand, we identified new hits, for further structural modifications, 3*H*-[1,2]dithiole-3-thiones, 3-(alkylthio) propylidene-3*H*-[1,2]dithioles, and [1,2]dithiolium iodide, as pharmacophores for *Tc*TIM and cruzipain inhibitors.

Further studies are needed in order to optimize the chemical structure and propose these systems as scaffolds for drugs against Chagas disease.
